# Supplementary material for: Within-host microevolution of Streptococcus pneumoniae is rapid and adaptive during natural colonisation
Source: Nat Commun. 2020 Jul 10;11:3442. doi: 10.1038/s41467-020-17327-w (PMC7351774; doi:10.1038/s41467-020-17327-w)
Supplement: Supplementary file 1 — Supplementary Information [file 41467_2020_17327_MOESM1_ESM.pdf]

Within-host microevolution of *Streptococcus pneumoniae* is rapid and adaptive during natural colonisation

Chaguza C. *et al*

Supplementary Table 1. Genetic diversity between strains of same serotype but different ST during colonisation.

Infant ID	Episode	Sequence type of strain		Number of SNPs		
		Strain 1	Strain 2	Minimum	Maximum	Mean
7	INF7:15B/C:1	ST4033	ST11776	1452	1458	1455
16	INF16:6B:1	ST5516	ST11689	552	642	596
19	INF19:11A:1	ST10968	ST5902	1036	1043	1040
33	INF33:19F:1	ST925	ST12308	1172	1221	1188
44	INF44:6A:1	ST913	ST5734	17735	18731	18113
50	INF50:19A:1	ST12377	ST847	17366	17366	17366
51	INF51:19A:2	ST847	ST10542	18819	18908	18864
58	INF58:19A:1	ST847	ST4029	19191	19214	19200
61	INF61:11A:2	ST5902	ST12309	24	132	41
72	INF72:19A:2	ST11691	ST847	19248	19288	19269
73	INF73:9L:1	ST12799	ST11705	4	22	9
81	INF81:10A:1	ST11690	ST5521	154	170	165
84	INF84:19A:1	ST1735	ST12316	469	469	469
92	INF92:19A:1	ST847	ST12191	17	28	23

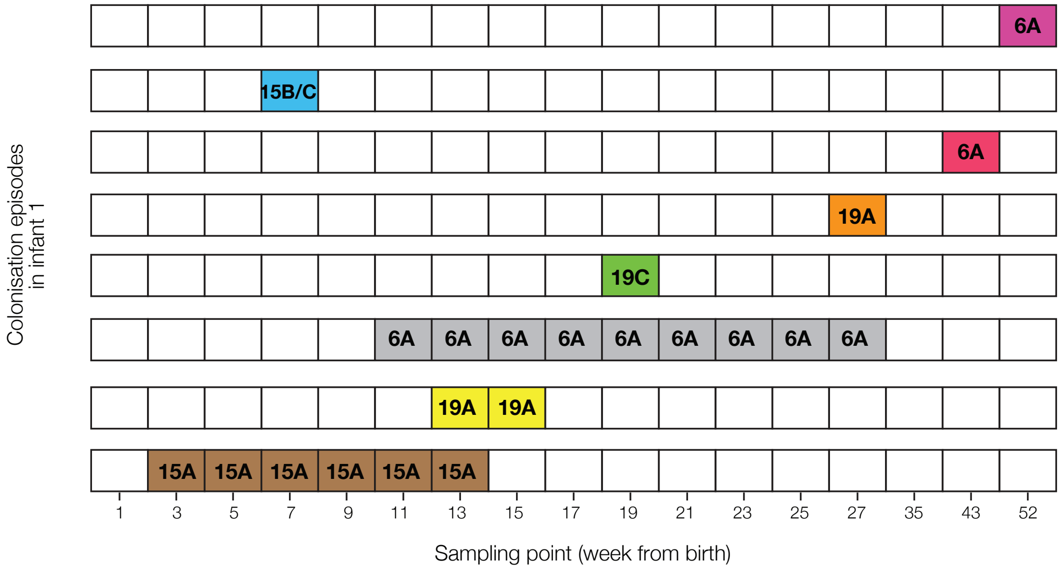

Supplementary Fig. 1. Definition of colonisation episodes. Colonisation episodes were assumed to start after either first detection or re-acquisition of a serotype and cleared when two consecutive culture-negative sample for the serotype were found for samples collected from week 1 to 27 from birth, while for samples collected after week 27 clearance was considered to have occurred when a single culture-negative sample for the serotype was detected.

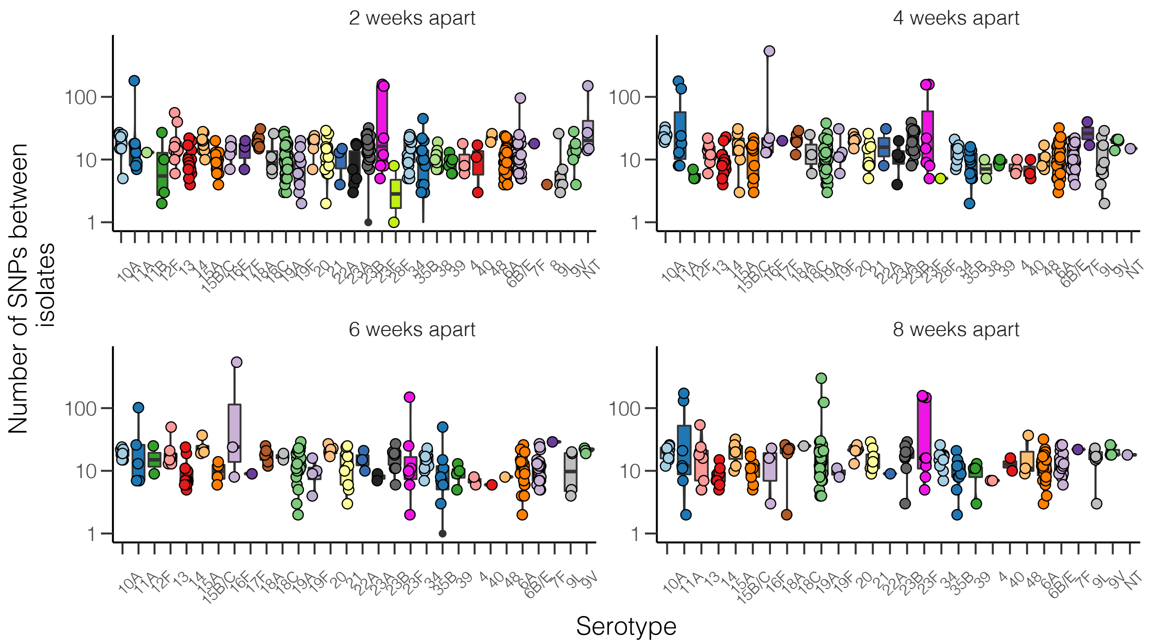

Supplementary Fig. 2. Within-host genetic diversity during natural colonisation. The strip charts show the number of SNPs calculated between isolates of the same serotype and ST within the same episode. The isolates sampled at 2, 4, 6 and 8 weeks from birth of an infant. The Y-axis of each plot is shown in $\log_{10}$ scale for clarity.

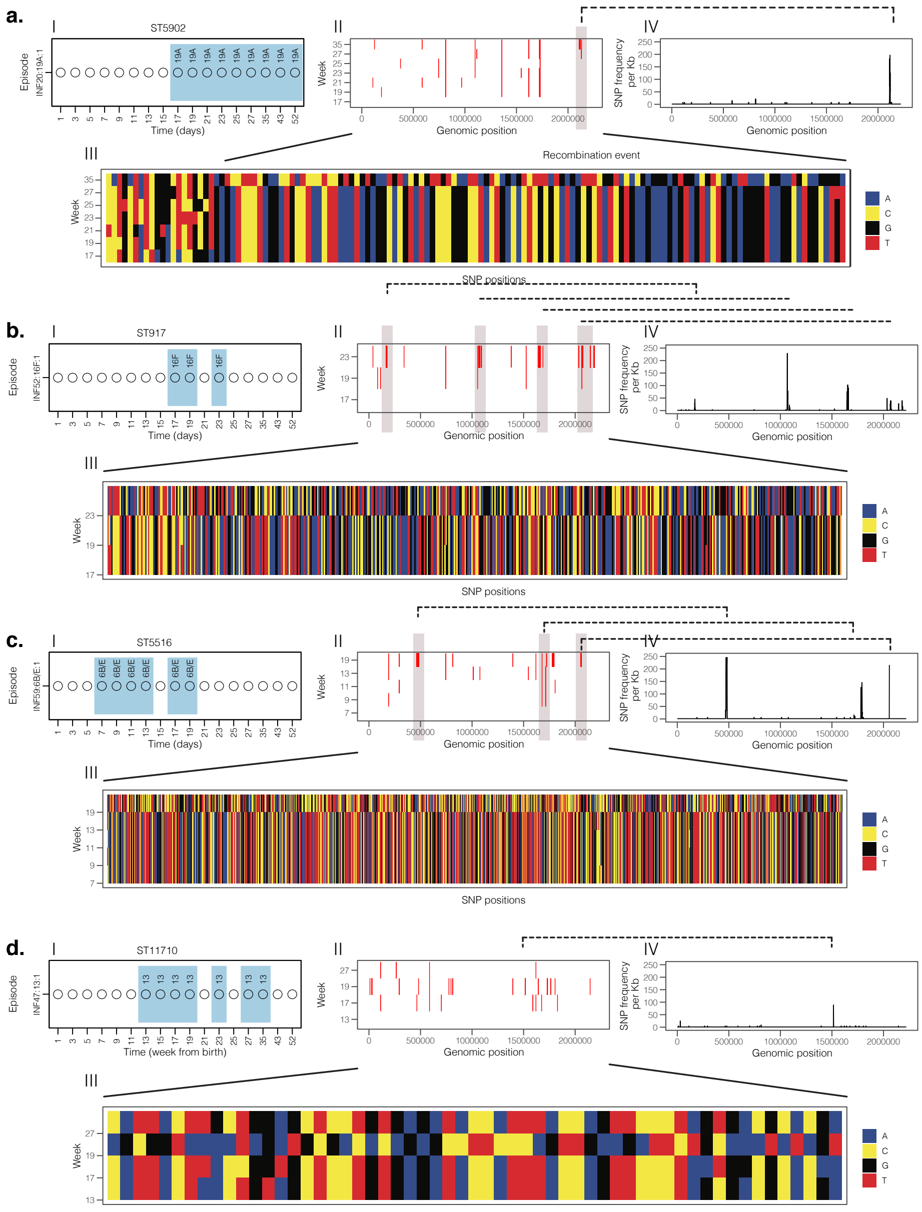

Supplementary Fig. 3. Within-host homologous recombination during natural colonisation. Panels **(a-c)** shows three examples of colonisation episodes where recombination events were detected. The episode name is shown in the format A:B:C where A, B and C represents the infant ID, serotype and number of episodes with the serotype respectively. (I) Colonisation episode showing the time points at which the serotype in the episode was detected. (II) Distribution of SNPs across genome. The coloured line (red) shows occurrence of a SNP in the strain using the first sequenced genome in the episode as the reference or ancestral strain. The SNP are enhanced for clarity. (III) A multiple sequence alignment of showing location of the SNPs and visual evidence of the emergence of a recombinant strain within the episode. The value for *r/m* represents the number of SNPs within recombination blocks relative to SNPs outside the blocks. (IV) The distribution of the SNPs is highlighted by the frequency polygon, generated using widow size of 1000bp, which shows spikes in the SNP density across the recombinogenic regions.

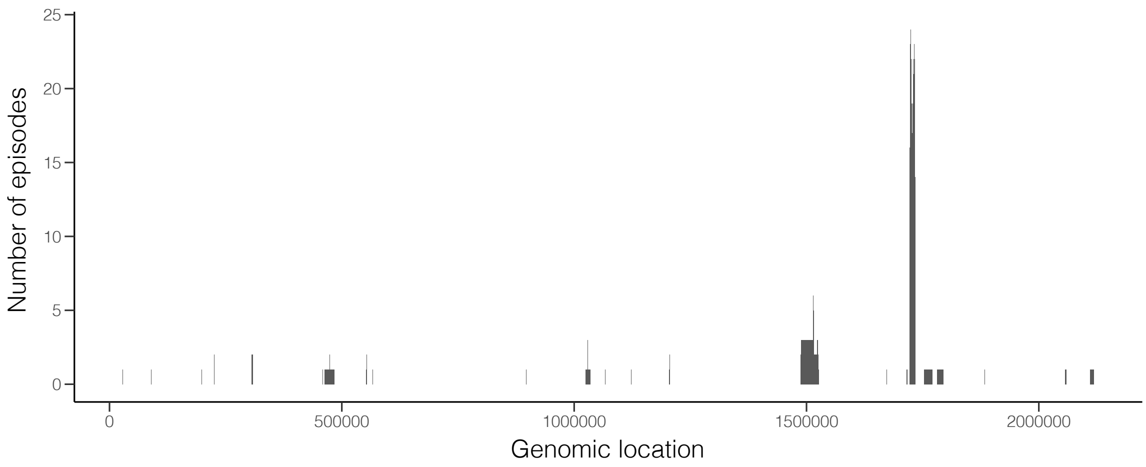

Supplementary Fig. 4. Hotspots of within-host recombination during natural colonisation. Maximum number of unique overlapping recombination events detected in different colonisation episodes are shown on the y-axis while the x-axis represent the position in the reference pneumococcal genome ATCC700669 (GenBank accession: NC_011900).

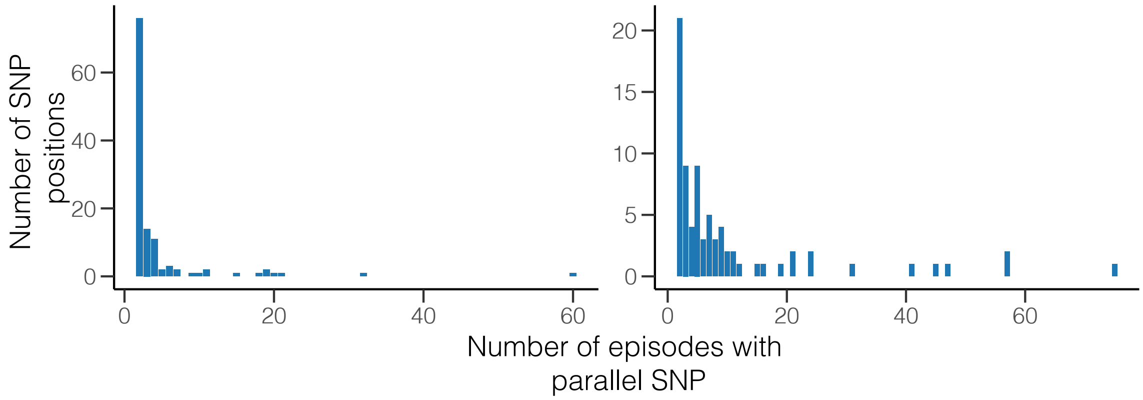

Supplementary Fig. 5. Histogram showing the distribution of parallel SNPs in genic and intergenic regions.

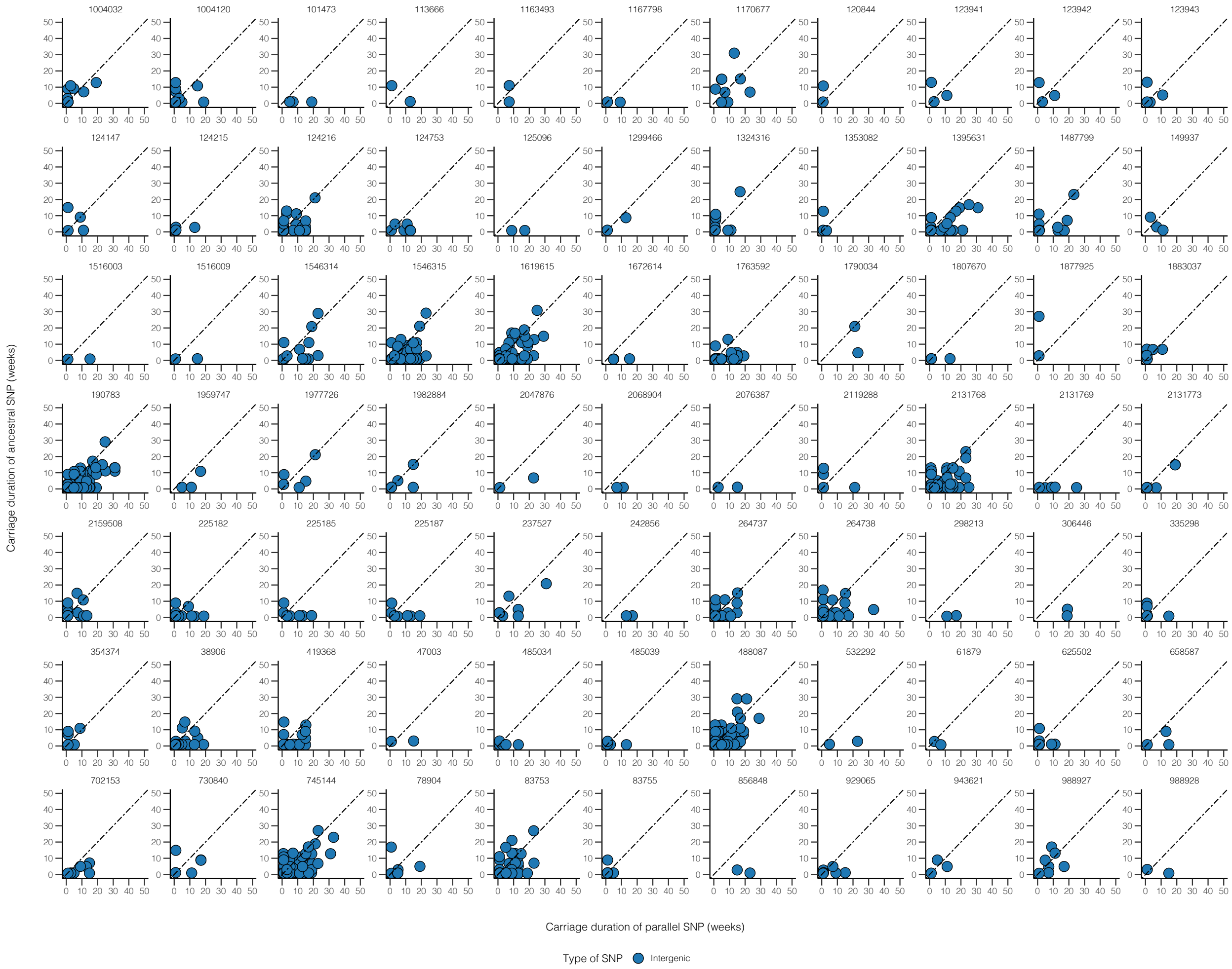

Supplementary Fig. 6. Scatter plots showing duration of carriage for the ancestral and parallel SNPs detected in intergenic regions during natural colonisation.

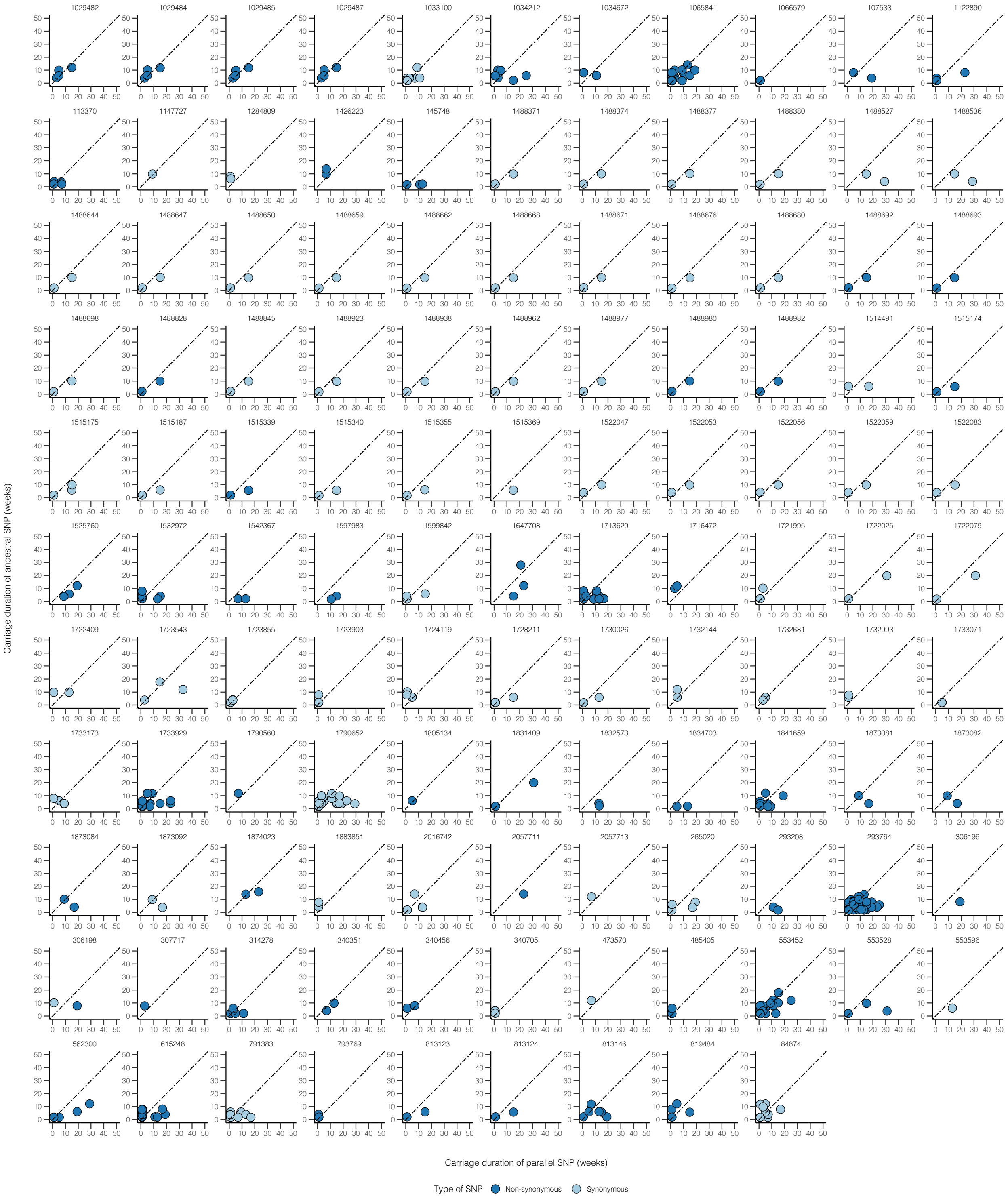

Supplementary Fig. 7. Scatter plots showing duration of carriage for the ancestral and parallel SNPs detected in genic regions during natural colonisation.
