# Supplementary material for: Within-host microevolution of Streptococcus pneumoniae is rapid and adaptive during natural colonisation
Source: Nat Commun. 2020 Jul 10;11:3442. doi: 10.1038/s41467-020-17327-w (PMC7351774; doi:10.1038/s41467-020-17327-w)
Supplement: Supplementary file 3 — Reporting Summary [file 41467_2020_17327_MOESM3_ESM.pdf]

nature research

Corresponding author(s): Chrispin Chaguza

Last updated by author(s): Jun 18, 2020

Reporting Summary

Nature Research wishes to improve the reproducibility of the work that we publish. This form provides structure for consistency and transparency in reporting. For further information on Nature Research policies, see Authors & Referees and the Editorial Policy Checklist .

Statistics

For all statistical analyses, confirm that the following items are present in the figure legend, table legend, main text, or Methods section.

n/a	Confirmed	
☐	☒	The exact sample size (*n*) for each experimental group/condition, given as a discrete number and unit of measurement
☐	☒	A statement on whether measurements were taken from distinct samples or whether the same sample was measured repeatedly
☐	☒	The statistical test(s) used AND whether they are one- or two-sided<br>*Only common tests should be described solely by name; describe more complex techniques in the Methods section.*
☐	☒	A description of all covariates tested
☐	☒	A description of any assumptions or corrections, such as tests of normality and adjustment for multiple comparisons
☐	☒	A full description of the statistical parameters including central tendency (e.g. means) or other basic estimates (e.g. regression coefficient) AND variation (e.g. standard deviation) or associated estimates of uncertainty (e.g. confidence intervals)
☐	☒	For null hypothesis testing, the test statistic (e.g. *F*, *t*, *r*) with confidence intervals, effect sizes, degrees of freedom and *P* value noted<br>*Give P values as exact values whenever suitable.*
☒	☐	For Bayesian analysis, information on the choice of priors and Markov chain Monte Carlo settings
☒	☐	For hierarchical and complex designs, identification of the appropriate level for tests and full reporting of outcomes
☒	☐	Estimates of effect sizes (e.g. Cohen's *d*, Pearson's *r*), indicating how they were calculated

Our web collection on statistics for biologists contains articles on many of the points above.

Software and code

Policy information about availability of computer code

Data collection	No software was used for data collection. All the samples used in this study were collected by trained clinicians and processed by experienced molecular microbiology team. The extracted DNA underwent whole genome sequencing and the resulting data was analysed using the open source tools listed below.
Data analysis	All software used in the analysis are freely and publicly available. Multilocus sequence typing (MLST) was done using MLSTCheck v2.0.1510612 (https://github.com/sanger-pathogens/mlst_check). Read mapping was done using SMALT v0.5.8 (www.sanger.ac.uk/science/tools/smalt-0). Variable nucleotide sites were identified from the consensus whole genome multiple sequence alignment from the mapping using SNP-sites v2.3.1 (https://github.com/sanger-pathogens/snp-sites). The distance matrix for the number of SNPs between genomes was generated using snp-dists v0.6.3 (https://github.com/tseemann/snp-dists). Data filtering and visualisation was performed using ggplot2 v3.1.0 (https://cran.r-project.org/web/packages/ggplot2/index.html). We detected homologous recombination events using Gubbins v1.1.1 (https://github.com/sanger-pathogens/gubbins) and multiple sequence alignment plots were created using alignfigR v0.1.1 (https://github.com/sjspielman/alignfigR). Genome-based serotyping was done using SeroBA v1.0.0 (https://github.com/sanger-pathogens/seroba). Multistate modelling for intermittently observed data was performed using msm v1.6.7 (https://cran.r-project.org/web/packages/msm/index.html) while statistical analysis was carried out in R v3.5.3 (https://cran.r-project.org/). Maps for the Gambia were generated using ggmap v3.0.0 (https://cran.r-project.org/web/packages/ggmap/). Genomic sequence data was processed using BioPython v1.7.6 (https://biopython.org/). Multiple sequence alignments diagrams were generated using alignfigR v0.1.1 (https://github.com/sjspielman/alignfigR). Three dimensional scatter plots were generated using scatter3D function in plot3D v1.3 package (https://cran.r-project.org/web/packages/plot3D/). Functional analyses of the genes using eggNOG-mapper v2.0 (http://eggnog-mapper.embl.de/). Insertion and deletions were realigned using GATK v4.0.3.0 (https://gatk.broadinstitute.org/hc/en-us).

For manuscripts utilizing custom algorithms or software that are central to the research but not yet described in published literature, software must be made available to editors/reviewers. We strongly encourage code deposition in a community repository (e.g. GitHub). See the Nature Research guidelines for submitting code & software for further information.

Data

Policy information about availability of data

All manuscripts must include a data availability statement. This statement should provide the following information, where applicable:

- Accession codes, unique identifiers, or web links for publicly available datasets
- A list of figures that have associated raw data
- A description of any restrictions on data availability

The whole genome sequences (reads) were deposited into the European Nucleotide Archive (ENA) and are publicly available under the accession numbers provided in Supplementary Data 1 of this paper. The reference genome sequence used for the read mapping (Genbank accession: NC_011900) is available from GenBank (https://www.ncbi.nlm.nih.gov/nuccore/NC_011900). The authors declare that all other data supporting the findings of this study are available within the paper and its supplementary information files.

Field-specific reporting

Please select the one below that is the best fit for your research. If you are not sure, read the appropriate sections before making your selection.

☒ Life sciences ☐ Behavioural & social sciences ☐ Ecological, evolutionary & environmental sciences

For a reference copy of the document with all sections, see nature.com/documents/nr-reporting-summary-flat.pdf

Life sciences study design

All studies must disclose on these points even when the disclosure is negative.

Sample size	The sample size calculation was based on infant nasopharyngeal microbiome data from a pilot study in the Gambia [Kwambana-Adams B et al. Sci Rep. 2017;7(1):8127. Published 2017 Aug 15. doi:10.1038/s41598-017-08717-0]. It was determined that a sample size of 90 (30 infants each of 3 groups) would give 90% power (p=0.05) to detect a difference of 25% or more in the mean number of bacterial genera carried in the nasopharynx 150 days after birth among the in infants in the three study groups. Participants were recruited on a roll-in basis until each group had at least 30 infants. A total of 102 infants were recruited in total and 96 completed the study. The infants were recruited within 7 days of birth and followed up bi-weekly for the first 6 months and bi-monthly thereafter until twelve months. A total of 1595 nasopharyngeal swabs were collected from the infants.
Data exclusions	Four infants died or left the study before 2 months and their data are excluded from the study.
Replication	Replication was not done for this study. Longitudinal studies of this magnitude are costly and require a lot of time to conduct especially in our study setting i.e. Sub Saharan Africa (the Gambia).
Randomization	Newborns were recruited from 27 villages with estimated birth rates between three and twenty-six per year. The villages were split into 3 groups of 9 villages with estimated population sizes of 2000 persons and birth rates of approximately eighty per year. Group I and II villages had to be at least 1Km from Group III villages where PCV-7 had been trialled. Group III villages were PCV naive. Trained village reporters in each village recorded and reported pregnancies, births, deaths and other serious events to the field team. To avert recruiting bias, participants were enrolled on a roll-in basis, whereby infants born in any of the participating villages and for whom written informed consent was granted were included in the study.
Blinding	No blinding was performed in this study. Blinding was considered not to be necessary for our longitudinal cohort study as the nature of the study was observational and no intervention was given to the newborn infants. We followed up all the enrolled infants and collected nasopharyngeal samples at specific time points to study strain dynamics, genomic diversity and evolution during natural colonisation therefore there was no risk for bias by not blinding the investigators.

Reporting for specific materials, systems and methods

We require information from authors about some types of materials, experimental systems and methods used in many studies. Here, indicate whether each material, system or method listed is relevant to your study. If you are not sure if a list item applies to your research, read the appropriate section before selecting a response.

Materials & experimental systems

n/a	Involved in the study	
☒	☐	Antibodies
☒	☐	Eukaryotic cell lines
☒	☐	Palaeontology
☒	☐	Animals and other organisms
☐	☒	Human research participants
☒	☐	Clinical data

Methods

n/a	Involved in the study	
☒	☐	ChIP-seq
☒	☐	Flow cytometry
☒	☐	MRI-based neuroimaging

Human research participants

Policy information about studies involving human research participants

Population characteristics	A total of 102 Gambian newborns were recruited within 7 days of birth and were followed up for twelve months (until they were one year old). Half (52/102) were born at home and all were born vaginally and all the infants were breastfed. The average birth weight was 3.1Kg. Most of the participants belonged to the Jola (58%) and Mandika (38%) ethnic groups and 51% (52/102) were males. A total of 3 infants died of post-natal complications, prior to PCV-7 vaccination at 8 weeks.
Recruitment	Newborns were recruited from 27 villages with estimated birth rates between three and twenty-six per year. The villages were split into 3 groups of 9 villages with estimated population sizes of 2000 persons and birth rates of approximately eighty per year. Group I and II villages had to be at least 1Km from Group III villages where PCV-7 had been trialled. Group III villages were PCV naive. Trained village reporters in each village recorded and reported pregnancies, births, deaths and other serious events to the field team. To avert recruiting bias, participants were enrolled on a roll-in basis, whereby infants born in any of the participating villages and for whom written informed consent was granted were included in the study.
Ethics oversight	The study was approved by the Joint MRC/Gambia Government Research Ethics Committee (SCC1108)

Note that full information on the approval of the study protocol must also be provided in the manuscript.
