# Supplementary material for: Within-host microevolution of Streptococcus pneumoniae is rapid and adaptive during natural colonisation
Source: Nat Commun. 2020 Jul 10;11:3442. doi: 10.1038/s41467-020-17327-w (PMC7351774; doi:10.1038/s41467-020-17327-w)
Supplement: Supplementary file 4 — Description of Additional Supplementary Files [file 41467_2020_17327_MOESM4_ESM.pdf]

Description of Additional Supplementary Files

File Name: Supplementary Data 1
Description: Characteristics of the study isolates from infants (Excel file).

File Name: Supplementary Data 2
Description: Summary of the colonisation episodes detected for each infant (Excel file).

File Name: Supplementary Data 3
Description: Summary of recombination events detected during extended colonisation episodes (Excel file).

File Name: Supplementary Data 4
Description: Description of genes identified in genomic regions containing recombination events (Excel file).

File Name: Supplementary Data 5
Description: Summary of parallel and non-parallel SNPs in genic and intergenic regions detected during different colonisation episodes (Excel file).

File Name: Supplementary Data 6
Description: Detailed summary of parallel SNPs detected during colonisation (Excel file).

File Name: Supplementary Data 7
Description: Number of SNPs detected per gene during colonisation (Excel file).

File Name: Supplementary Data 8
Description: Number of synonymous and non-synonymous SNPs detected per gene during colonisation (Excel file).
